# Perspectives of clinicians on the potential impact of social media on social and emotional wellbeing, training needs, and screening recommendations: a preliminary examination

**DOI:** 10.3389/fpsyt.2025.1484456

**Published:** 2025-02-27

**Authors:** Sarah E. Domoff, Stacey B. Armstrong, Heide Rollings, Amy Mancuso, Carol A. Janney

**Affiliations:** ^1^ Department of Psychology, University at Albany, Albany, NY, United States; ^2^ College of Social Work, The Ohio State University, Columbus, OH, United States; ^3^ Graduate Medical Education Department, Pine Rest Christian Mental Health Services, Grand Rapids, MI, United States; ^4^ Division of Psychiatry and Behavioral Medicine, College of Human Medicine, Michigan State University, East Lansing, MI, United States; ^5^ Grants Department, Pine Rest Christian Mental Health Services, Grand Rapids, MI, United States; ^6^ College of Human Medicine, Michigan State University, East Lansing, MI, United States; ^7^ Department of Epidemiology and Biostatistics, College of Human Medicine, Michigan State University, East Lansing, MI, United States; ^8^ Center for Statistical Consultation and Research, University of Michigan, Ann Arbor, MI, United States

**Keywords:** adolescents, social media, cyberbullying, problematic media use, sextortion

## Abstract

**Introduction:**

Social media is integral to adolescents’ lives, with the separation between adolescents’ online and offline worlds harder to distinguish. Adolescent development occurs online via the opportunity to connect with others and explore themselves. Despite the potential for benefits, some adolescents with underlying mental health conditions are at risk for stressful online experiences, such as cyberbullying. The complexity of the impact of social media on adolescents necessitates an understanding of mental health providers’ perspectives on their observations of youth treated, how they support youth in navigating social media, and suggestions for clinical and research priorities to address barriers encountered in developing resilient and prosocial interactions online. The purpose of this qualitative study was to understand these perspectives in stakeholders across mental health-related disciplines.

**Methods:**

In Fall 2022 to Spring 2023, 14 participants were interviewed regarding their perspectives on social media and mental health, how they screen for harmful experiences online, and their approach to supporting youth who have experienced online stressors. We used an inductive thematic analysis to identify themes.

**Results:**

Themes reflected clinicians’ perceptions of positive and negative impacts of social media on youth; how they communicate with youth about their experiences; preferences or recommendations for screening for harmful experiences; and barriers encountered in addressing use and impacts.

**Discussion:**

We outline recommendations for implementing screening for social media experiences, responding to harmful online experiences, and future clinical research directions to fill gaps in training and service provision related to adolescents’ social media use.

## Introduction

In the United States, most adolescents own a smartphone by age 14 (1). With greater access to smartphones comes increased access to social media and consumption. A recent study shows that 35% of adolescents use the top five social media platforms “almost constantly,” with TikTok and Snapchat having the highest proportion of constant users (2). Furthermore, adolescent social media use is so integrated into daily life that 36% of adolescents say they spend too much time on social media, and 54% say it would be difficult to give it up (2).

Recent research prompted the U.S. Surgeon General to issue a warning regarding the potential risks associated with adolescent use of social media that warns of an association between social media use and mental health concerns (3). For instance, the amount of time spent using social media and problematic media use has been linked to internalizing problems (4, 5), such as depression (6), suicide (7, 8), and anxiety (5). Furthermore, the link between the decreased quality of sleep and social media use is notable (9), given the relationship between sleep quality and mental health outcomes (10).

Many of the negative outcomes associated with social media are related to the type of content that adolescents are engaging with. For instance, experiencing online victimization (often referred to as cyberbullying) is associated with depression (11), self-harm and suicidality (12, 13), and anxiety (14, 15). Another type of online victimization, called sextortion, has become a focus of research as social media and access to the internet have increased drastically over time. Sextortion is threatening the release of non-consensual, sexually explicit, intimate, or embarrassing images, typically to get more images, sexual acts, money, or something else (16). These events have been associated with several negative mental health outcomes, including depression (17, 18), anxiety (17), stress symptoms (19), and suicidal thoughts (17). Both online victimization and sextortion are associated with a number of negative mental health outcomes; however, it is important to note the directionality of these associations is unclear, especially among vulnerable adolescents (20).

While the negative outcomes associated with social media use are compelling, the findings in the literature are mixed (21, 22). In fact, there is growing evidence that some aspects of social media use may be beneficial. For instance, social media use has shown benefits in pertinent developmental tasks that are critical to healthy adolescent functioning, such as developing and maintaining friendships (18, 23) and safe identity exploration (18, 24). Leveraging social media may be especially important among marginalized adolescents, such as LGBTQ youth living in rural areas, where feelings of social isolation may be more common (25). Despite the association between social media use and depression, other research has identified “positive concurrent relationships” between social media use and self-esteem (26) and increased self-esteem (18, 24). Increased self-esteem may be linked to perceptions of social support (24), another identified benefit of online social networking.

Although online activities are an important aspect of adolescence, there is a lack of information on how mental healthcare providers perceive and address their youth clients’ online experiences (however, see Moreno et al. (2023) (27)for an innovative social media counseling program offered by primary care providers to youth). Prior research on U.S. mental healthcare providers’ awareness of the links between social media and mental health has focused on physicians-in-training (i.e., medical students) (28); other research on addressing youth social media use has been limited to providers in the United Kingdom (29, 30). Prior research among practitioners in the United Kingdom raise concerns about gaps in knowledge and training related to addressing online harm and digital risks experienced by youth. It is unclear how mental health care practitioners in the U.S. support young people in navigating social media and what clinical and research priorities can be established to address barriers to developing resilient and positive interactions online. Further, although prior research has identified clinicians’ perspectives on social media impacts and good practice indicators (31), few inquiries have considered screening and intervention practices of clinicians, and gaps pertaining to problematic social media use, online victimization, and sextortion. Given the call to action by the U.S. Surgeon General (3), the associated harms of problematic media use, and the ways social media can be leveraged to promote healthy adolescent development, we sought to understand clinicians’ and other service providers’ perspectives on problematic media use and its impacts on adolescent mental health, barriers to screening for online victimization, and advantages of social media use.

## Methods

### Procedure

This study was approved by the Michigan State University, College of Human Medicine IRB. Community stakeholders from youth-serving organizations and healthcare systems were invited to participate in semi-structured interviews about adolescent social media use and mental health. In Fall 2022, we sent emails to clinicians providing services to adolescents in a psychiatric healthcare organization or personnel in community agencies also serving adolescents with a range of mental health care needs across diverse settings. Email recipients were asked to share the request for research participation with clinicians or staff affiliated with their clinic, program, or organization (henceforth referred to as clinicians). The inclusion criteria were that the participants had to be at least 18 years old and to be actively working with youth. Individuals who expressed interest were scheduled to participate semi-structured interviews via virtual conferencing with an interviewer not involved with the reading of transcripts or theme generation. Interviews occurred from November 2022 through April 2023. A total of 13 interviews occurred with 14 participants (for convenience, one interview included two participants from the same organization). Informed consent was obtained from each of the participants, and no identifying information about the participants was linked to the transcripts of their interviews. After interview completion, the interviewer informed the participants that information gleaned from this study would be shared with them and would be used to inform future training and program development. Participants were compensated $40 for their time. The interviews lasted approximately 30 to 45 minutes.

### Participants

Fourteen participants from various training backgrounds participated in the interviews. The age of participants ranged from 24 years to 60 years (M = 42.0 years, SD = 11.1 years). The majority identified as White/Caucasian (n = 11, 78.6%), two (14.3%) identified as Black/African American and 1 (7.1%) identified as more than one race. Several respondents were clinicians in a hospital system (n = 5; 35.7%). The specializations of the MDs included psychiatry, adolescent medicine, and pediatrics. The other participants were employed in a community mental health system (n = 2; 14.3%); worked in a mental health system or a nonprofit mental health system providing outpatient services (n = 2; 14.3%); worked in the juvenile justice system (n = 2; 14.3%); worked for a health and human services agency (n = 2; 14.3%) or worked in private practice (n = 1; 7.1%). The participants were from one region in the Midwest (i.e., Western or Southwestern Michigan).

### Semi-structured interview questions

Questions posed were open-ended and the interviewer followed up with probes to gain a deeper understanding of the clinicians’ perspectives and for elaboration. These questions were generated based on input from psychiatrists and other mental health care providers regarding concerns on how to best address or incorporate social media experiences of adolescents during treatment. Please see **Table 1** for a list of the interview questions.

**Table 1:** Semi-structured interview questions and probes.

Interview questions	Potential follow-up probes
What stressful experiences do adolescents have through their social media or internet use?	Tell me about other stressful experiences
What positive experiences do adolescents have through their social media or internet use?	Tell me about other positive experiences
How do you learn about adolescents’ use of social media and online communication?	Do you ask about their social media use? Why/why not?
How do you learn about adolescents’ experiences of cyberbullying and other types of negative online social interactions?	Do you ask about this? Why/why not?
If an adolescent came to you with an experience of cyberbullying or other types of negative online social interactions (e.g., online victimization), what strategy/strategies would you suggest they use to address the issue?	Follow up about strategies used for experiences of sexting or sextortion
Tell us about experiences that professionals working at your organization have had with adolescents experiencing of cyberbullying or other types of negative online social interactions (e.g., online victimization).	Follow up about experiences of sexting or sextortion
How frequently and in what situations do you think youth should be screened for types of negative online social interactions (e.g., online victimization)?	Follow up about screening for sextortion
What do you perceive are the benefits and drawbacks of screening for negative online social interactions (e.g., online victimization)?	Follow up about screening for sextortion
What concerns or fears do you think would prevent youth from disclosing events of online victimization?	Follow up disclosing about sextortion
What advice or words of wisdom do you have for people working with youth regarding social media and online experiences?	

### Qualitative analyses

After interviews were transcribed and checked for accuracy, three readers from distinct clinical training backgrounds (psychiatry, psychology, and social work) conducted the inductive thematic analysis. All transcripts were read and initial codes were generated by the readers to inform their identified themes across the interviews. Readers approached the analysis using a realist method at the semantic level (40). The realist method entails denoting the lived experiences or reality of the participants; in identifying themes in the transcripts at the semantic level, readers focused on the explicit content shared by participants (i.e., did not make inferences beyond what was stated). The readers identified a theme if the belief, experience, or perspective occurred across interviews from multiple participants; importance of the theme or its extent of discussion was not impacted by the number of participants who expressed the content described by the theme (as such, no frequency coding was conducted with this type of analysis).

## Results

Eight themes were identified, which reflect clinician perceptions of positive and negative experiences on social media for youth with whom they work; experiences with how they communicate with youth about their experiences; preferences or recommendations for screening for harmful social media experiences; and barriers that clinicians and staff experience in addressing social media use and impacts. Below we describe each theme and provide illustrative quotes (additional examples provided in **Table 2**).

**Table 2:** Supplemental quotes.

Theme	Illustrative Quotes
1. Cyber-victimization is the primary online stressor that youth experience	“I feel for the youth that I've worked with, what makes it big is when their safety is in jeopardy. When someone talks about coming to their home or fighting them.”
	“The first one that comes to mind is cyberbullying. It gives a platform where people feel slightly anonymous and say things that they wouldn't necessarily say in in a public setting, but again, it's still causing pretty significant harm to the person receiving that information.”
2. Not getting likes, seeking validation, and impact on self-esteem emerged as additional negative aspects of social media use	“They're getting a lot less validation, whether it's likes or upvotes or messages to them. They are getting a lot less attention than their peers in everything is not monetized, but qualified. So, you can see if somebody has 155 likes or something like that, and then they might only get one. Also, just the sheer number of people that will respond or choose to not respond if it’s a more direct communication, like a text or they try to FaceTime someone like they're just not getting the response that they're aware that their peers are getting.”
3. Connecting with peers or friends stands out as a positive experience youth have on social media.	“I do think social media allows them the time, especially if you are in diverse areas, to connect with people and also a way for them to potentially try on different identities.”
	“So it's giving them the opportunity to connect with someone on a shared interest, like cast the wider net to find their people.”
4. Organic discussion of social media experiences comes up in sessions	“The concern part comes from parents. They'll share: ‘I found them using this’ or ‘they were on this site’ or something along those lines. So I get the negative from the parental side…”
5. Adolescents’ willingness to disclose may be shaped by fear of embarrassment or getting in trouble	“A lot of times it's like, ‘well, I told my teacher. But then they just made fun of me for telling the teachers’ and things like that. So, it's like the fear that telling an adult could make the bullying worse or it could just make the perpetrators better at hiding it or that you're letting get worse by telling on the individual.”
	“They may be afraid they’d get their phone taken away.”
	“I think the fear of being exposed, the fear of everyone knowing that they did engage. If they did send something and them seeing it, and the fear of getting into trouble.”
	“That if they engage willingly, that they should be in trouble.”
6. Therapists should be asking about social media at intake and throughout treatment.	“Yes. I could see having a question or two. I mean, there's so many things that we're screening for trauma abuse, neglect, suicidal ideation. You know the substance use and then all the mental health symptoms. So, I could see it as part of an intake for therapy, it would be a natural question to ask with the clinical interview when you're doing an intake.”
	“Yes, absolutely. They should ask about it. I would say for me, the therapist should ask more frequently than the doctor... I think almost during every session, your initial check in or when you're following up, you're asking if they feel safe at home or if they feel safe in their environment. Why not ask? If they feel safe online as well.”
7. Pediatricians should be asking about social media via screening at well child visits	“I think for a primary care a pediatrician’s office, it's part of their routine physicals and checkups and doctor's offices are already kind of integrating the PHQ and asking about general abuse, suicidal thoughts or behaviors. I could see it fitting into that kind of general emotional health and well-being and safety questions and the same goes for cyberbullying and for sextortion.”
	“So we should be asking it. I would say at the pediatrician’s office, we screen for depression and anxiety as well.”
8. Do not have a clear process for handling sextortion or cyber-bullying	“I'm not sure what to do.”
	“I don't know that I have an answer for that one. I think it happens more often than we realize because they aren't openly going to talk about sex…”
	“When there is bullying-- the frustration of, like, what's the school's role? What's the parents’ role? What's the parents of the other kids’ role? It becomes this like finger pointing situation of who's responsible for the bullying and how to stop it.”

### Perceptions of adolescents’ experiences on social media

Three themes emerged related to clinicians’ perceptions of adolescents’ experiences on social media. The first theme was that cyber-victimization is the primary online stressor that youth experience. Clinicians stated:

”The other thing is my clients are higher risk for being targets so sometimes the parents will explain these really long situations that happened and at times again they’re being bullied online, as well as in-person.”

“I would say one of the things I deal with quite often is like cyberbullying. So kids picking on other children through social media.”

“I would say the biggest things I see is probably online bullying stuff about feeling there’s rumors about them or hearing degrading comments from friends or other people online. ”

Clinicians identified that another negative aspect of social media was how it may impact their clients' self-esteem (e.g., not getting likes and seeking validation through social media).

“I see a lot of my clients doing comparisons between themselves and what they see on social media, leading to feelings of depression, feelings of less than, and doing a lot of reaching out.”

“There’s lots of pro eating disorder platforms and websites. Even though we’re seeing greater turn towards body positivity, it doesn’t always reach our younger population in that same way.”

Despite the perceived negative impacts, clinicians noted that connecting with peers or friends stands out as a positive experience youth have on social media. Example quotes for theme 3 include:

“I see it helps a lot of my clients feel a sense of connection. They feel some sort of community, whether that be them following a lot of people who do the artwork that they like, or the rappers that they like, they feel some sort of sense of connection within their following and it leads to a sense of self-concept, some higher self-esteem feeling as though they have a purpose and like the community.”

“I think social connection. I think there are some groups of teens, I’m thinking of the LGBTQ plus community, for example, where they might be able to connect with other individuals and various places throughout the world who have the same shared lived experience and might feel safe for them to connect with these individuals in ways that they might not feel as connected to that community and their everyday life.”

### Communication with adolescents about their social media experiences

Clinicians detailed how communication with their adolescent clients occurred regarding both positive and negative social media experiences. First, clinicians generally expressed that organic discussion of social media experiences comes up in sessions; they do not use pre-planned screening or direct questioning of these experiences (Theme 4). Illustrative quotes include:

“Now it’s just such a pervasive part. I don’t think you can really talk to them without it coming up.”

“I would say the issues with cyberbullying and online are talked about altogether just naturally. I think our clients bring it up and talk about it.”

“I think it comes up more organically. I don’t ask anything that specific. If it’s something that’s part of what they fill out on their paperwork or something is listed that’s related to social media, then I will be more intentional about asking about social media use. Other than that, it just tends to come up.”

Theme 5. Adolescents’ willingness to disclose may be shaped by fear of embarrassment or getting in trouble. Clinicians noted that screening for harmful online experiences may be complicated by adolescents’ concerns about consequences.

“Shame and guilt or fear getting in trouble, or adolescents have a general notion, especially again the ones that are higher functioning that I think are aware that taking pictures of themselves nude is considered child pornography and they themselves are afraid of getting in trouble, even if they’re the victims that they wouldn’t want to be legal ramifications against them.”

“I think the fear of being exposed, the fear of everyone knowing that they did engage. If they did send something and them seeing it, and the fear of getting into trouble. I have a lot of girls who will come in and not understand that there is some protection for minors, but because they engage, they feel as though they are the perpetrator and the one that could get charges and not necessarily get help.”

### Preferences or recommendations for screening for social media experiences

Theme 6. Given the myriad of experiences that adolescents have online, clinicians shared that therapists should be asking about social media at intake and throughout treatment.

“I think we as providers and therapists should be asking … I never thought about it, but I probably should have really have a standard questionnaire about how much time does your kids spend on social media, what kinds of things do they see, do you know what they see or who they’re talking to? That should be something we are regularly screening for because it is our kid’s worlds, unfortunately.”

“I would say, as long as you’re under the age of 18, they should be asked about it, no matter what. How frequently is hard, though I would say maybe at minimum once a month just to check in. Like ‘hey, do you feel safe online?’

“I think that’s actually a great question to have on an intake document. So they’re filling out their new patient paperwork, and especially for our teen population to have a question that simply says, have you experienced cyberbullying? A yes or no is all it takes for us to then ask about it more in session, which then puts it on our radar to periodically check in.”

Theme 7. In addition to mental health care providers routinely screening for cybervictimization, clinicians also thought other medical professionals should be checking for online stressors. In particular, clinicians expressed that pediatricians should be asking about social media via screening at well child visits.

“I could also see it at the annual pediatricians’ appointments that might be a natural time to ask, ‘have you ever had this experience?’”

“I think those questions should be asked or when they’re doing a wellness check at their doctor’s office. They could start asking some depression and anxiety questions. Well, maybe this would be a great one to use as a screener for teen clients as well.”

“I could also see it at the annual pediatricians’ appointments that might be a natural time to ask, have you ever had this experience? Has anyone ever propositioned you to send a picture or how would you, you know, kind of.”

### Barriers experienced in addressing social media use and impact

Theme 8. Clinicians indicated gaps in clinical protocols and therapeutic intervention strategies when adolescents disclose harmful social media/online experiences. Clinicians expressed not having a clear process for handling sextortion or cyber-bullying disclosures (beyond establishing safety and telling parents).

“Or it’s hard to nail down who’s responsible for it. Like, is it the school that needs to step in, or is it the parent that has to awkwardly call the parent of the other kids and try to engage in some conversation? So that’s kind of more what I’m experiencing.”

“So, as you know, part of our job is keeping kids safe and risk and nailing down. So, you know, there’s like a really clear process in place for abuse and neglect. We call our number; we report it and then we move on our way. But with this, it’s so tricky. What’s the age of the person trying to get this from? There’s just so many … where is this person physically located? Is this even a real person that’s doing it? I think that would be so hard.”

“If it was another, I mean, I can imagine in certain situations getting legal authorities involved. But again, I think it could also be hard…”

## Discussion

This article describes the perceptions of the impact of social media use on youth mental health through qualitative interviews with multiple clinicians who work with youth in various treatment settings. The study found that social media holds a significant risk to youth mental health, especially due to cybervictimization and comparisons with peers. There are also benefits that can aid mental health through connection with online communities. Clinicians identified the need for a centralized resource to identify clearer processes for frequency and method in assessing risk and guidance in responding when youth disclose concerns.

Clinicians recognized that adolescents perceive some aspects of social media usage as advantageous and beneficial. They believe it helps foster social connections and find individuals who share common interests, especially on niche or unique topics that may be shared less in their social network. This finding is consistent with prior research identifying the advantages of social media (18, 23, 25). Clinicians also identified that their adolescent patients encounter adverse experiences on social media, including cyberbullying, feelings of inadequacy from constant comparisons, as well as concerns about getting validation of their content from others. These findings are consistent with youths reporting concern about their safety, including cyberbullying, while using social media (13, 15, 32). Clinicians appear to have a balanced perspective on both the risks and benefits of social media use among their clients.

Clinicians identified that conversations about experiences on social media come up unprompted for clinicians directly in their work with youth experiencing mental health challenges. However, for more harmful content, like cyberbullying, despite the frequency with which these experiences occur (33), they are less likely to be brought up directly by youth. This finding contrasts with research suggesting that utilizing social support from adults is an effective coping strategy for cyber victimization (34). It may be that youth are hesitant to reach out for help regarding cyberbullying; and specific screening questionnaires should be utilized to ask about cyberbullying to more routinely identify individuals in need (35).

Clinicians in this study identified the importance of routine screening for adolescents’ problematic media use in primary care settings. This result is consistent with the American Psychological Association’s health advisory recommendations (36). Similarly, the American Academy of Pediatrics updated their HEADS3 psychosocial screening to HEADS4 in 2018 to include social media (37), given the importance of pediatricians screening youth for their social media experiences. In addition to other social media experiences, this screening includes the question: Have you personally experienced cyberbullying, sexting, or an online user asking to have sexual relations with you? (37)

Clinicians in this study identified that behavioral health professionals, such as therapists, should similarly be screening for youth’s social media use, including cyberbullying and sextortion. Due to the nature of the therapeutic relationship and frequency of visits, the clinicians involved in this study suggested that behavioral health professionals should make more frequent and thorough inquiries about their patients’ social media usage than primary care physicians. National organizational guidance, including those such as APA (36), identifies that adolescents should be routinely screened for problematic media use, though they do not give enough guidance to clarify the role specific to behavioral health professionals.

The clinicians in this study demonstrated a significant gap in clinical knowledge concerning the protocols and appropriate steps to follow when a patient discloses instances of cyberbullying or sextortion. When reviewing accessible resources and guidance online, most are targeted at school professionals, parents, or youth themselves (38). There are fewer available resources directed toward behavioral health professionals. General guidance on managing cyberbullying includes supporting youth through identifying bullying, keeping records of the events and saving information such as screenshots related to cyberbullying events, reporting to online service providers as cyberbullying often violates the terms of service established by social media sites and internet providers, and recognizing when cyberbullying is reportable to law enforcement and schools. Several states have responded to cyberbullying with legislation focusing on prevention, intervention, and consequences, though these have not been thoroughly explored in research (35).

### Clinical implications

Based on clinician feedback in this study, it is recommended that clinical conversations about social media use occur organically rather than through structured interviews, with the exception of screening for cyberbullying and sextortion. As mentioned previously, resources that may be useful for screening for cyberbullying and sextortion include the HEADS4, which has five questions specific to social media use, including one specific to online victimization (37). Assessment for problematic social media use should also occur at annual well-child visits. Additionally, clinicians should assess social media use both at intake and throughout treatment. Finally, resources relevant to coping with cyberbullying and managing social media use in healthy ways could be useful for behavioral health clinicians. One such resource is the American Academy of Pediatrics Center of Excellence on Social Media and Youth Mental Health (39).

### Study limitations

This study was limited by a small sample size and a narrow geographical region from which clinicians reflect. Examination of perspectives in other regions of the US and in other countries is needed to inform generalizability. Further, given the time-consuming nature of an hour-long interview, it is possible that clinicians with the most clinical or practice demands were not able to make time in their schedule to participate. Clinicians deeply immersed in clinical activities may have additional and important perspectives that were not captured in this analysis.

### Future research directions

Future research on the topic of social media and youth mental health is necessary to better understand the nuances of both the positive and negative aspects of social media use. This research would include a more thorough understanding of youth’s perspectives and youth-focused intervention to support them in navigating these complex situations to improve safety and mental health outcomes. Formative work on good practice indicators for a range of social media use practices has been identified among providers in the U.K. (31), which could be expanded to include harmful online experiences identified in this study. Clinician-based interventions should also be developed to support mental health clinicians in guiding youth and their families in best practices with regard to social media use and mitigation of harmful effects (similar to the work of Moreno et al., 2023 (27) with primary care provider training). In these interventions, it will be critical to engage and educate parents and caregivers as well, given their crucial role in supporting and navigating these complexities directly with their children and teens.

## Data Availability

The datasets presented in this article are not readily available because Participants did not consent to their interviews being shared. Requests to access the datasets should be directed to Heide.rollings@pinerest.org.
